# Impact of pneumococcal vaccine response on asthma exacerbation frequency in young children

**DOI:** 10.1002/iid3.331

**Published:** 2020-07-17

**Authors:** Claudia P. Eisenlohr, Esther M. Chartrand, Marta R. Barzaga, Miguel J. Lanz

**Affiliations:** ^1^ AAADRS Clinical Research Center Coral Gables Florida

**Keywords:** asthma exacerbations, childhood asthma, eosinophils, pneumococcal vaccination, *Streptococcal pneumonia*

## Abstract

**Introduction:**

Chronic asthma is a heterogeneous disease, and increased eosinophils have been shown to predict increased asthma exacerbations, especially in adults. Recent recommendations suggest the need for supplemental PPV‐23 vaccination in older children with chronic asthma.

**Methods:**

To investigate differences in preschool asthma, our case‐cohort study comprised of 127 children, mean age 47 months (32‐65), with a history of asthma exacerbations requiring more than three courses of systemic steroid bursts and more than six antibiotics courses in the previous year.

**Results:**

At baseline, mean antibody titer response to *Streptococcus pneumoniae* was decreased at 2.6 ± 2 out of 14 serotypes, despite prior complete pneumococcal conjugate vaccine (PCV) vaccinations. All children were readministered pneumococcal vaccinations with PCV‐13 booster and/or PPSV‐23. Mean postvaccination pneumococcal vaccine (PV) titer response was 16 ± 5 out of 23 serotypes. After contacting 91 parents/caretakers, 75 responded with less frequency of corticosteroids and antibiotic use for asthma exacerbations after PV. This group had baseline eosinophil counts of 211 ± 36/µL, while those without improvement were significantly higher at 371 ± 123/µL,*P < .05. There were no significant differences (*P* > .05) between the two groups from other baseline measures including demography or atopic status.

**Conclusions:**

This subset of children with exacerbation‐prone asthma had poor antibody titer response to *Streptococcus pneumoniae*, even with prior complete PCV‐13 immunization. Identification of low antibody responses to PV serotypes may provide a targeted therapeutic approach to reduce wheezing exacerbations in a precise asthma phenotype in children.

**Abbreviations**
CS: corticosteroids
PV: pneumococcal vaccine
PCV: pneumococcal conjugate vaccine
PPSV: pneumococcal polysaccharide vaccine

Childhood asthma is heterogeneous condition with multiple phenotypes, arising from interactions between genetics, infections, and environmental exposures. In children, 2 to 5 years of age, asthma phenotypes were originally classified into either transient early wheezing or persistent wheezing categories.^1^ Analysis of merged studies of preschool asthmatics have shown that with corticosteroid (CS) treatment certain phenotypes have divergent responses manifested with the probability of exacerbations.^2^ Phenotypes with minimal allergic sensitizations had more likelihood of asthma exacerbations with CS use.

Certain microbiome compositions in respiratory flora are responsible for asthma exacerbations early in life.^3^ Studies have shown that infants, who later developed asthma, had an immune response to *Streptococcus pneumoniae* with increased interleukin 5 (IL‐5) and IL‐13 and decreased IL‐17 and IL‐10.^4^ Further data in young children has shown that colonization with *S. pneumoniae* can contribute to the severity of asthma exacerbations with or without the presence of rhinovirus.^5^ Infant vaccination with pneumococcal conjugate vaccine 13 (PCV‐13) initiated in 2010 in the United States to replace the PCV‐7 vaccine. But there is scant data on the immunogenicity of pneumococci in preschool asthmatics. Despite having prior PCV vaccination, asthmatic children continue with higher risks of invasive pneumococcal disease than their counterparts. Vaccine studies administering PPSV‐23 after PCV‐13 showed improved antibody levels when boosted >10 months afterwards.^6^ Recent recommendations from a meta‐analysis of studies before 2011 suggested the need for supplemental PPV‐23 vaccination in asthmatic children.^7^

We proposed to evaluate the parent‐report rate of exacerbation‐prone preschool asthmatics with high frequency of systemic steroid bursts and/or antibiotic use. Second, we reviewed baseline tests to elucidate differences in asthma endotypes^8^ with exacerbation outcomes in this age group.

To investigate differences between treatment‐outcomes in preschool asthma in children aged 24 to 71 months, a case study from a 6‐year period in an asthma specialty clinic was performed. All children had a documented history by the clinician and parent of recurrent wheezing and asthma in the prior year. Inclusion criteria included the presence of wheezing exacerbations requiring ≥3 systemic CS bursts per year and/or respiratory infections requiring ≥6 courses of antibiotics per year, which was verified from medical records and parent recall. All prior treatments for asthma flare‐ups were prescribed by their referring primary care provider or emergency room/urgent care center. Children were excluded if they had taken systemic steroids or antibiotics in the previous 4 weeks or taken either for nonrespiratory etiologies, had known prior primary/secondary immunodeficiency, or had incomplete immunization schedules.

Due to the high frequency of exacerbations and antibiotic courses, these children had an allergy/immunology evaluation for allergen sensitivity and immune response. Respiratory testing was not performed due to their ages. Follow‐up contact with parents/caretakers to reassess frequency of asthma exacerbations and systemic steroids/antibiotics use >1 year postinitial evaluation/treatment was performed. This study, which included children managed with the current standard medical practice for asthma, was reviewed and exempted by the ethics/IRB committee of the Salus Institutional Review Board.

The cohort comprised of 127 children, mean age 47 (32‐65) *SD* months, 60% (76) male, with a history or presence of 55% (70) ≥3 CS/yr, 45%(57) ≥6 antibiotics/yr, 43% (54) indoor pets, 42% (53) eczema diagnosis, and 39% (50) parental asthma. Allergen sensitivity to ≥1 allergen by prick skin testing was positive in 48% (61). Mean baseline laboratory values revealed white blood corpuscles 9.0 × 10^3^/µL, IgE 163.0 IU/mL, IgG 796 mg/dL, IgA 87 mg/dL, IgM 97 mg·dL, and serum eosinophils 247/µL, which were all normal for age. Mean number of antibody titers with responses to Streptococcus pneumoniae (>1.3 mcg/mL) was decreased at 2.6 ± 2.3 out of 14 serotypes, which is noteworthy since all had been vaccinated with PCV‐13 as per reviewed immunization records. Poor specific‐antibody responses were found with serotypes 4, 9V(68), 1, and 18C(56): (95%;121, 89%;113, 86%;109, 85%;108), respectively.

All children were administered pneumococcal vaccinations (PV) with PCV‐13 booster and/or PPSV‐23. Postvaccination pneumococcal antibody titers had been obtained in a third of children, whom had a mean number of antibody titer response (>2 mcg/mL) to 16 ± 5 out of 23 serotypes. There were no significant differences between frequency of visits, prescribed controller therapy and environmental avoidance measures between groups with or without postvaccination titers.

There were 72% (91) completed contacts with parents/caretakers, and 82%(75) responded with less number of bursts/use of CS and antibiotics for wheezing/infectious asthma exacerbations after PV. The remaining responses had increased or same number of bursts/use of CS/antibiotics for asthma exacerbations after PV. Those with responses of improvement after PV had a mean baseline eosinophil count of 211 ± 36/µL, (eos% 2.4 ± 0.4), while those worsening or without improvement were significantly higher at 371 ± 123/µL, (eos% 4.8 ± 1.8), *P < .05 (Table 1). There were no other significant differences (*P* > .05) between demography, atopic status, including eczema, parental asthma, and allergen sensitization, and all other baseline laboratory measures between these groups of PV responders and nonresponders.

**Table 1:** Characteristics of postpneumococcal booster vaccine responders with improvements of decreased systemic steroids/antibiotic use compared to nonresponders with continued increased use

	Post‐PV responders vs nonresponders		
Demographics			
Number	75	16	
Age, mean, months	54	53	
Males, %	60	75	
Positive PST/in vitro tests (%)	42	66	
Baseline (n), 95% CI			
WBC, ×10^3^/mcL	9.2 ± 2.8	9.2 ± 2.9	n.s.
Eosinophils, %	2.4 ± 0.4	4.8 ± 1.8	*P <* .05^a^
Eosinophils, serum, /mcL	211 ± 36	371 ± 123	*P < *.05^a^
IgG total, mg/dL	833 ± 173	836 ± 179	n.s.
IgA total, mg/dL	91 ± 41	95 ± 47	n.s.
IgM total, mg/dL	100 ± 46	87 ± 36	n.s.
IgE total, IU/mL	97 ± 128	96 ± 83	n.s.
*S. pneumoniae* titers (n > 1.3 mcg/mL)	2.4 ± 0.6	3.9 ± 2.5	n.s.

Abbreviations: PST, prick skin testing; PV: pneumococcal vaccination.

a *P*  < 0.05, serum eos and %.

Our study aimed to identify endotypes in young asthmatics that relate to differences in subsequent treatment responses as measured by their exacerbation rate. First, those with higher absolute/percent eosinophil counts consistent with eosinophilic asthma was predictive for continuing high frequency of exacerbations than those with lower eosinophils or infection‐prone asthma. Second, these results are unique in the literature because despite prior complete PCV vaccinations, this subset of asthmatics with exacerbation‐prone wheezing had poor antibody titer responses to *S. pneumoniae*, even with prior complete PCV‐13 immunization schedules during infancy. This data suggests booster PV immunization and the subsequent rise in antibody titers can reduce asthma morbidity by decreasing systemic CS bursts and antibiotic use.

Although the low specific PCV serotypes 1, 4, 9V(68), 18C(56) found in our results have been documented to have increased invasive potential,^9^, ^10^ larger specific PV serotype studies into causal relationships of these serotyped‐infections with asthma exacerbations are required. Limitations of these results are that it is a case‐control, single‐site study, and without a control group. Future prospective‐designed studies would be recommended.

Young children with recurrent asthma exacerbations requiring frequent CS bursts and/or antibiotics should be evaluated for low functional antibody responses by their asthma specialist. Identification of young asthmatics with lower eosinophil counts may benefit from this immune evaluation to reduce exacerbations related to infectious triggers. This identification of low specific‐antibody responses to PV serotypes may provide a targeted therapeutic approach to reduce wheezing exacerbations in a precise asthma phenotype in children.

## AUTHOR CONTRIBUTIONS

All listed authors have made substantial contributions to conception/design, acquisition of data, analysis of data, and have been involved in drafting the manuscript or critically revising for content. All authors have given final approval of the version to be published, taken responsibility for the content, and agreed to be accountable and ensure accuracy of the work.

## CONFLICT OF INTERESTS

MJL research funding from AstraZeneca, Genentech, Optinose, Stallergens; and consulting fees/honorarium from AstraZeneca, Novartis, Optinose, Sanofi Regeneron. CPE, EMC, and MRB declare that there are no conflict of interests.

## ETHICS STATEMENT

This study was performed as an audit of standard clinical practices and reviewed by the Salus Institutional Review Board.

## Data Availability

The data that support the findings of this study are available from the corresponding author upon reasonable request.
